# Acute methotrexate toxicity in a patient with psoriasis: a case report

**DOI:** 10.11604/pamj.2024.47.19.39012

**Published:** 2024-01-17

**Authors:** Hind Abouzahir, Ahmed Belhouss, Hicham Benyaich

**Affiliations:** 1 Medico-Legal Institute, Ibn Rochd University Hospital, Casablanca, Morocco,; 2 University Hassan II, Faculty of Medicine and Pharmacy, Casablanca, Morocco

**Keywords:** Medical liability, methotrexate toxicity, case report

## Abstract

Aside from rheumatoid arthritis, methotrexate is also used to treat cancer, psoriasis, and other diseases. Side effects with methotrexate are possible, as they are with any medication. This drug is extremely potent and has the potential to produce serious adverse effects. Those who use this medication need to be tracked often. We provide a case of a patient with psoriasis vulgaris who died due to methotrexate administration without proper dosage verification. A female patient in her forties had a history of psoriasis vulgaris of the lower limbs. Under treatment, she developed acute methotrexate toxicity. This drug was taken as an intramuscular injection per day in an infirmary without checking that the dose regimen prescribed was per week. She developed extensive bullous and pustular lesions associated with digestive signs related to generalized toxiderma. But at that point, she had septic shock, which led to her death a few weeks after the methotrexate injection. The medical responsibilities of the doctor, pharmacist, and nurse were discussed. To conclude, methotrexate is not a killer drug in most cases, but it can be extremely harmful if it's overused. Acute toxicity is a potentially fatal condition, and a deeper understanding of its potential toxicity is still necessary.

## Introduction

Methotrexate (MTX) is an antimitotic cytotoxic antagonist of folic acid. It is a cytostatic medication that is utilized as an anticancer agent in the treatment of cancer and other malignancies. It suppresses the immune system and reduces inflammation when taken in low doses [1]. Psoriasis has been treated with low doses of MTX for more than half a century since it is both an effective and safe medication. The principal form of elimination is through the kidneys, and the rate of this process is determined by both the dosage and the way in which it is taken [1]. Even at a low therapeutic dose, serious side effects can occur, including hepatic cirrhosis, pulmonary fibrosis, impaired renal function, erythema of the skin, and vasculitis. In order to properly diagnose acute MTX poisoning, this necessitates vigilant monitoring of renal function tests and blood counts, in addition to vigilantly scanning for mucosal sores or ulcerations in the skin. If the guidelines are not followed, there is a risk of severe toxicity, which could possibly result in death [2]. There are many articles that talk about adverse outcomes from MTX, and even fewer of those don't talk about fatalities that occurred because this medicine is so secure. Here, we report a case of a patient with psoriasis vulgaris who died of an MTX overdose without checking the dose prescribed.

## Patient and observation

**Patient information:** a female patient in her forties who had a medical history of psoriasis vulgaris of the lower limbs for 5 years under regular treatment. She was treated with oral retinoids. She had aggravation of lesions, and then her treatment was switched to MTX (15 mg per week). However, following an error in the dispensing of the prescribed packaging of methotrexate by a pharmacy, the patient received a dose of 15 mg of MTX per day through intramuscular injection in a nursing facility without verifying the prescribed dosage.

**Clinical findings and therapeutic interventions:** the patient was admitted to the hospital with a fever and extensive bullous and pustular lesions associated with digestive signs related to generalized toxiderma that appeared on the third day after receiving a third intramuscular injection of MTX. Blood tests showed pancytopenia (hemoglobin 10.5 grams, white blood cells 800 cells/mm^3^, and platelet count 100,000 cells/mm^3^) with normal renal and liver functions.

**Follow-up and outcomes:** her state evolution was complicated by rectorragia, melena, and dyspnea with persistant fever in a shock septic state. Despite the resuscitation measures, she died a few weeks after the MTX injection due to multiorgan failure.

**Autopsy and histopathological findings:** a medicolegal autopsy was requested by the prosecutor for suspicion of medical malpractice.

**Figure 1:**
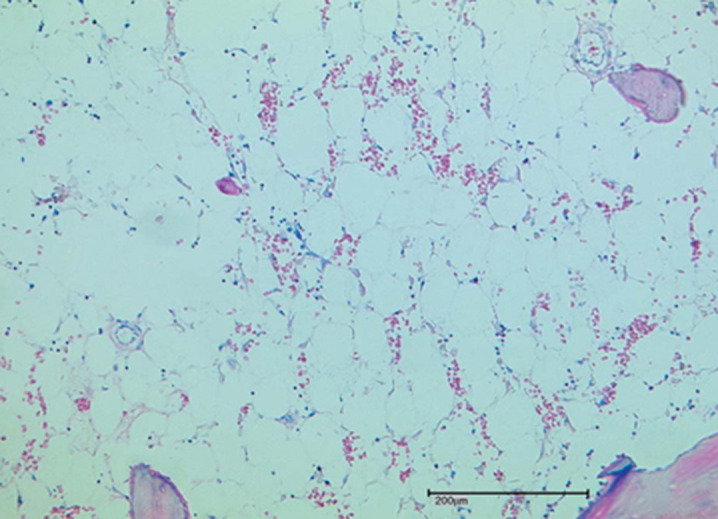
bone marrow hypocellularity after methotrexate injection

External examination of the corpse found pustular and bullous skin lesions in the thoraco-abdominal, back, and limbs, with impetiginized erosions covering the face. An autopsy revealed moderate sero-hematic pleural effusion and congestion of most organs, without any other specific lesions. Bone marrow samples were taken for histopathology examination, and the results showed cellular hypoplasia without neoplastic changes (Figure 1).

**Ethical approval/informed consent:** this article does not contain any studies with human participants or animals performed by any of the authors. The information was collected and kept confidential during data entry, data analysis, communication, and publication of results. Anonymity was respected and the name of the deceased was not mentioned.

**Informed consent statement:** informed consent was obtained from the deceased’s family.

## Discussion

Methotrexate has become an important drug in the treatment of numerous chronic pathologies such as rheumatoid arthritis, psoriasis, lupus, Crohn's disease, and other diseases. The side effects of this medication are very serious, mainly affecting the bone marrow, liver, intestines, kidneys, lungs, skin, and blood [3].

Patients who suffer from severe forms of psoriasis typically receive treatment with methotrexate administered in low doses. It is a preferred option due to its therapeutic value, known side effect profile, and inexpensive cost, and it is typically recommended as a weekly dose. On the other hand, it has the potential to be extremely poisonous and even lethal [4]. Despite the fact that patients ingesting high dosages are more likely to experience toxicity, every dosing plan has the potential to cause toxicity [4]. Across the globe, there have been a number of documented deaths linked to the use of methotrexate. Patients have frequently taken methotrexate on a daily basis rather than once every seven days as a result of errors that were committed by the patient, the clinician, or the pharmacist. The most recent deadly incidents in New Zealand were recorded in 2006 and 2012, respectively [5].

A study of four different Danish registers identified 173 errors, with nearly two-thirds resulting in harm. Serious harm occurred in 15%, and there were 9 deaths (5%). These involved incorrect daily administration (31%), dosing errors (62%), and improper monitoring (9%). Serious outcomes, including deaths, were more common when hospital physicians made mistakes in prescribing involving daily administration rather than weekly administration [6,7]. In our case, taking MTX as a daily dose instead of a per-week dose was the most common cause of acute MTX toxicity. Pneumonitis, which may happen following a single dose of methotrexate, is the most common reason for death resulting from the use of methotrexate. Bone marrow suppression is another cause of mortality with multiple organ failure [8-10], and it occurred in our reported case.

In our case, the pharmacist disregarded the rules by handing over methotrexate without explanation and, moreover, did not comply with the doctor's prescription. Whereas the nurse carried out the MTX injection as a daily dose. Also, she should have contacted the prescribing clinician to check with him on the appropriate dose of this medication when there is doubt. All participants in our case were responsible: the dermatologist because of his prescription, which was lacking instructions with the dose; the pharmacist who didn´t explain the prescription; and the nurse who did not check how many times the prescribed dose should be taken.

However, the appeals court retained the responsibility of the dermatologist, who made a mistake by not writing clear instructions with the dose in his prescription. The court considered that the medical malpractice committed by the doctor made the patient lose her chance of survival. Consequently, the dermatologist was ordered to compensate the family of the deceased by providing financial restitution.

## Conclusion

This study serves as a valuable reminder of the importance of recognizing and managing acute methotrexate toxicity in patients with psoriasis. By fostering increased awareness, implementing appropriate monitoring strategies, and tailoring treatment regimens, healthcare providers can mitigate the risk of adverse events and ensure safe and effective therapeutic outcomes for individuals with psoriasis receiving methotrexate therapy.
